# Comparative Evaluation of *Pseudomonas aeruginosa* Adhesion to a Poly-(2-Methacryloyloxyethyl Phosphorylcholine)-Modified Silicone Hydrogel Contact Lens

**DOI:** 10.3390/vision7010027

**Published:** 2023-03-21

**Authors:** Valerie Harris, Reed Pifer, Paul Shannon, Monica Crary

**Affiliations:** Alcon Research, LLC, Fort Worth, TX 76134, USA

**Keywords:** *Pseudomonas aeruginosa*, keratitis, ocular infection, contact lenses, medical device, 2-methacryloyloxyethyl phosphorylcholine, MPC, surface treatment, bacterial adherence, microbial adhesion

## Abstract

*Pseudomonas aeruginosa* is the most common causative agent associated with microbial keratitis. During contact lens wear, pathogens may be introduced into the ocular environment, which might cause adverse events. Lehfilcon A is a recently developed contact lens with a water gradient surface composed of polymeric 2-methacryloyloxyethyl phosphorylcholine (MPC). MPC is re-ported to impart anti-biofouling properties onto modified substrates. Therefore, in this in vitro experimental study, we tested the capability of lehfilcon A to resist adhesion by *P. aeruginosa*. Quantitative bacterial adhesion assays using five strains of *P. aeruginosa* were conducted to compare the adherence properties of lehfilcon A to five currently marketed silicone hydrogel (SiHy) contact lenses (comfilcon A, fanfilcon A, senofilcon A, senofilcon C, and samfilcon A). Compared to lehfilcon A, we observed 26.7 ± 8.8 times (*p* = 0.0028) more *P. aeruginosa* binding to comfilcon A, 30.0 ± 10.8 times (*p* = 0.0038) more binding to fanfilcon A, 18.2 ± 6.2 times (*p* = 0.0034) more binding to senofilcon A, 13.6 ± 3.9 times (*p* = 0.0019) more binding to senofilcon C, and 29.5 ± 11.8 times (*p* = 0.0057) more binding to samfilcon A. These results demonstrate that, for various strains of *P. aeruginosa*, lehfilcon A reduces bacterial adhesion compared to other contact lens materials.

## 1. Introduction

*Pseudomonas aeruginosa* is a common Gram-negative bacterial pathogen that can detrimentally affect multiple organ systems. *P. aeruginosa* is a leading cause of ocular infections, including conjunctivitis, dacryocystitis, keratitis, and corneal ulcerations [1,2,3,4,5,6,7]. Keratitis caused by *P. aeruginosa* is characterized by severe pain, photophobia, generalized redness, swelling, and discharge. The disease can progress to corneal ulceration, scaring, and in severe circumstances, loss of vision [8]. Unfortunately, contact lens usage is associated with the development of microbial keratitis (CLMK) [2,7,9,10]. *P. aeruginosa* is the microorganism most frequently associated with CLMK [10]. Eyecare professionals and contact lens manufacturers offer recommendations to patients for the safe use of contact lenses. However, patients routinely fail to follow recommended guidelines for contact lens usage [11,12,13,14,15,16,17]. Poor contact lens hygiene can circumvent the microbiological safeguards required for safe usage, setting the stage for infection. A likely contributing mechanism for CLMK occurs when an inadequately disinfected contact lens serves as a vector for the introduction of a pathogen into the eye. Contact lens storage cases are intended to serve as cleaning vessels when paired with biocide-containing contact lens care (CLC) solutions. Historically, there have been keratitis outbreaks believed to be linked to inadequate CLC disinfection efficacy under some circumstances. These include outbreaks of the comparatively rare pathogens *Fusarium solani* [18,19] and *Acanthamoeba* [20]. It is widely understood, and now codified in ISO 18259, that the efficacy of multipurpose disinfection solutions (MPDS) can depend upon the materials of the contact lens, contact lens storage case, and the presence of soiling agents [21,22,23,24,25]. However, additional disinfection variability can arise when real-world patient practices, such as reusing or topping off contact lens care solution, dilute the effective concentration of biocides.

Unfortunately, pathogens such as *P. aeruginosa* can express or acquire a wide variety of resistance genes and biofilm-promoting pathways that enable survival when challenged with an insufficient biocide concentration [26]. In a study by Subedi et al., *P. aeruginosa* strains isolated from keratitis patients were challenged with various dilutions of multipurpose disinfection solutions (MPDSs) [27]. The authors found, for multiple strains, substantial variations between formulations in their minimum bactericidal concentrations. A similar study by Khan et al. observed strain-to-strain variations in the susceptibility of *P. aeruginosa* isolates to MPDS formulations and constituent biocides [28]. In addition to genetically driven differences in disinfection efficacy between organisms, microbial life cycle can play an important role in determining MPDS susceptibility. Biofilms formed by *P. aeruginosa* can be particularly resistant to disinfection by MPDS formulations [29]. Biofilms can form within contact lens storage cases, and the efficiency of removal depends upon the cleaning regimen [30]. Once established, a reservoir of live microbes within the storage case may then serve as a source for constant reinoculation of the eye. Indeed, *P. aeruginosa* contamination of storage cases is associated with keratitis [31,32].

Disinfection by MPDS depends upon the identity and concentration of the biocide, the context in which the agent is applied, and the genetics and behavior of the organism being targeted. Thus, susceptibility to disinfection by biocides in MPDS is analogous to antibiotic efficacy, which is similarly context-dependent. As with clinical antibiotic interventions, it is inevitable that there will be disinfection failures. The consequences of a disinfection failure ultimately depend upon the interactions of the infectious organisms and the patient. The ocular surface is constantly challenged with microorganisms that do not cause apparent disease. The epithelial barrier and tear-film components generally prevent infection [33,34,35,36]. However, pathogenic organisms such as *P. aeruginosa* can express a variety of virulence factors that enable corneal tissue degradation, including secreted proteases [36,37,38] and type III secreted effector proteins [39,40]. Similarly, *P. aeruginosa* can exhibit disease-promoting phenotypes such as motility [41,42] and biofilm formation [43].

The pattern of medical-device-associated infections is not unique to CLMK and is similar to other those of susceptible organ systems, such as catheter-associated urinary tract infections (CAUTI) and ventilator-associated pneumonia (VAP). Unlike CAUTI and VAP, prevention of CLMK is dependent upon continual compliance with good hygienic practices by contact lens wearers rather than by medical professionals. Therefore, new mitigation strategies for reducing the infection risks posed by contact lens usage should include multiple layers of redundancy to account for user non-compliance.

SiHy contact lenses provide patients with the advantage of high oxygen permeability, which reduces the epithelial effects of corneal hypoxia [44]. However, siloxane-based materials of silicone hydrogels are natively hydrophobic. Poor wettability of contact lenses is associated with patient discomfort and biomass deposition, including microbial adhesion [45]. Therefore, a variety of strategies are used to decrease the hydrophobicity of SiHy contact lens surfaces, including plasma treatment, copolymerization with hydrophilic molecules, and surface coatings. Lehfilcon A is a recently developed SiHy contact lens material that is surface modified with poly-(2-methacryloyloxyethyl phosphorylcholine) (PMPC) [46,47]. MPC is remarkable for its anti-biofouling properties and has been shown to be effective on a multitude of substrates [47,48,49,50,51,52,53,54]. Previously, lehfilcon A was demonstrated to reduce protein and lipid deposition compared to its silicon hydrogel base material [46,47]. Recently, we demonstrated that lehfilcon A showed reduced adhesion by the ocular pathogen *Serratia marcescens* [55]. Therefore, we explored the degree to which the poor adhesion of *S. marcescens* to lehfilcon A extends to the pathogen *P. aeruginosa*. In this manuscript, we compare the anti-biofouling performance of lehfilcon A to a panel of SiHy contact lens materials when challenged with multiple *P. aeruginosa* strains.

## 2. Materials and Methods

### 2.1. Strains and Materials

*P. aeruginosa* strain ATCC 10145 was obtained from the American Type Culture Collection. The *P. aeruginosa* strain CL79, isolated from a contact lens case, and the keratitis-associated strains 6294 and 6206, were contributed by Suzanne Fleiszig [56]. GSU#3, derived from a human corneal ulcer, was contributed by Donald Ahearn [57].

*P. aeruginosa* strains were maintained on trypticase soy agar (TSA) slants with incubation at 30–35 °C for 16–24 h. For assays, cells were grown on fresh slants and resuspended in phosphate buffered saline (PBS). Cell densities were then adjusted to a 10^6^–10^7^ colony forming units (CFU)/mL using spectrophotometry. CFU quantifications were performed by dilution plating on TSA supplemented with 0.5% polysorbate 80 and 0.07% lecithin (MCTA), followed by incubation at 30–35 °C for 18–24 h. Duplicate plate counts were averaged. The eluted concentration of *P. aeruginosa* (CFU/mL) was calculated by multiplying the averaged plate count by the dilution factor used in plating.

Contact lenses were commercially acquired for experiments. Contact lenses tested were lehfilcon A (TOTAL30™, Alcon, Fort Worth, TX, USA), comfilcon A (Biofinity^®^, Cooper Vision^®^, Scottsville, NY, USA), fanfilcon A (Vitality^®^, Cooper Vision^®^, Scottsville, NY, USA), senofilcon A (Acuvue^®^ Oasys, Johnson & Johnson Vision Care, Jacksonville, FL, USA), senofilcon C (Acuvue^®^ Vita™, Johnson & Johnson Vision Care, Jacksonville, FL, USA), and samfilcon A (Ultra^®^, Bausch + Lomb, Rochester, NY, USA). To begin experiments, contact lenses were removed from their packaging using sterile forceps and equilibrated overnight in PBS.

### 2.2. Confocal Microscopy

Fluorescently labeled *P. aeruginosa* ATCC 10145 was prepared to facilitate visualization of bacterial interactions with contact lenses according to the procedure described in Pifer et al. with minor modifications [55]. Briefly, TSA slants were prepared as described above and harvested into PBS, and the cells were pelleted. Cell pellets were rinsed twice with PBS and resuspended in a buffer composed of nine parts PBS and one part 7.5% sodium bicarbonate. A 10 mg/mL solution of 5(6)-carboxytetramethylrhodamine succinimidyl ester (TAMRA-SE, Life Technologies, Eugene, OR, USA) was prepared in DMSO and mixed with cells to achieve a concentration of 0.1 mg/mL. The staining reaction was carried out for 10 min at 30–35 °C. The staining procedure was repeated once, followed by five sequential rinses with PBS to remove excess stain. For adhesion experiments, stained cells were resuspended to 10^6^–10^7^ CFU/mL in PBS and incubated in a 12-well plate in the presence of contact lenses previously equilibrated in PBS. Negative control lenses were exposed to sterile PBS. Stained cells were left in contact with contact lenses for 2 h with gentle agitation at 100 RPM at 30–35 °C in an Innova 40R shaking incubator. Non-adherent cells were rinsed from the lens by gently transferring each lens into wells containing fresh PBS and agitating for 1 min at 30–35 °C, 100 RPM. Three sequential rinses were performed. Cells adhered to contact lenses were then fixed with 4% paraformaldehyde in PBS, followed by an additional rinse in PBS to remove excess paraformaldehyde. Contact lenses were suspended in Prolong Live antifade reagent for imaging. Images were collected using a Nikon A1R confocal microscope at 4.975 micrometer resolution at 10X magnification. A 561 nm laser was used for excitation, and emission spectra were collected from 570 to 616 nm.

### 2.3. Adhesion Assays

Quantitative adhesion assays were performed to evaluate the density of *P. aeruginosa* adhered to contact lens surfaces. Equilibrated contact lenses were transferred into 12-well plates containing *P. aeruginosa* at 10^6^–10^7^ CFU/mL in PBS. *P. aeruginosa* adhered to the contact lenses for 2 h at 30–35 °C during agitation at 100 RPM. Three sequential 1 min rinses in PBS were performed at 30–35 °C, 100 RPM. Lenses were then transferred into 10 mL PBS supplemented with 0.05% polysorbate 80 and vigorously vortexed for 2 min to elute *P. aeruginosa*. Live organism recovery from each lens was quantified by dilution plating, as described above. A schematic of this procedure was created with BioRender.com.

### 2.4. Statistical Analysis

Contact lens surface areas were calculated as described in Pifer et al. [55] Briefly, surface area in square millimeters was computed as:

A = 2π[(D/2)^2^ + S^2^], (1)

where the contact lens diameter is represented by D. The posterior sag (S) is computed using the contact lens diameter and base curve (BCE):

S = BCE − √ (BCE^2^ − (D/2)^2^). (2)

Following adhesion assay recovery, the eluted concentration of *P. aeruginosa* (CFU/mL) was multiplied by the 10 mL recovery volume to determine the absolute recovery per lens (CFU/lens). The density of *P. aeruginosa* on a contact lens was then calculated by dividing the absolute recovery per lens by the surface area of the contact lens (CFU/mm^2^). The log_10_ microbial density was then calculated for each lens (log CFU/mm^2^).

Pooled mean fold differences in adhesion were calculated to compare contact lens material performance for multiple *P. aeruginosa* strains. To accomplish this, the ratio of the average microbial density (CFU/mm^2^) of each lens material to that of lehfilcon A was calculated. This yielded the fold difference in adhesion between a comparator material and lehfilcon A. The fold differences in adhesion for each strain were averaged to calculate the pooled mean fold differences in adhesion among *P. aeruginosa* strains.

Statistical analyses were performed with GraphPad Prism version 9.2.0. To determine if the microbial recovery was significantly different between contact lenses, 2-tailed t-tests were run on the log microbial densities to compare datasets without assuming equal sample variance. For each analysis, Bonferroni correction was used to adjust the initial significance level of α = 0.05 to account for multiple comparisons.

## 3. Results

**Figure 1:**
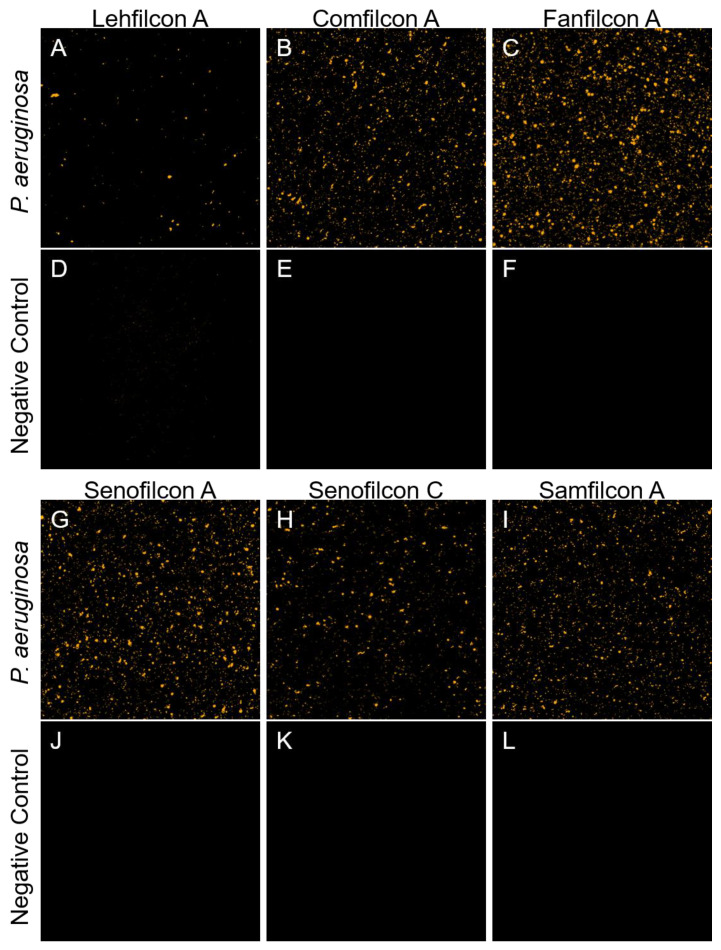
Qualitative assessment of *P. aeruginosa’s* adherence to soft contact lenses. Confocal microscopy of contact lenses treated with TAMRA-stained *P. aeruginosa* strain ATCC 10145 (**A**–**C**,**G**–**I**) or mock treated with sterile PBS (**D**–**F**,**J**–**L**). Bacteria are visible as foci attached to the surface of lehfilcon A (**A**), comfilcon A (**B**), fanfilcon A (**C**), senofilcon A (**D**), senofilcon C (**E**), or samfilcon A (**F**). A 0.42 × 0.42 mm field of view from the center of each contact lens is shown.

To visualize how *P. aeruginosa* interacts with the surfaces of a contact lenses, we fluorescently labeled *P. aeruginosa* strain ATCC 10145 with TAMRA-SE, a dye that forms covalent bonds with primary amine residues of proteins. We performed confocal microscopy to qualitatively evaluate the population of *P. aeruginosa* that was bound to the surface of a panel of silicon hydrogel contact lenses currently available on the market, as of 2023. We observed that comparatively low amounts of ATCC 10145 bound to lehfilcon A (Figure 1A) relative to comfilcon A (Figure 1B), fanfilcon A (Figure 1C), senofilcon A (Figure 1G), senofilcon C (Figure 1H), and samfilcon A (Figure 1I). Uninfected control materials (Figure 1D–F,J–L) showed considerably lower fluorescent signals, as expected. This outcome is similar to our observation of low accumulation of *S. marcescens* on lehfilcon A [55]. We also observed a trend of relatively low adhesion on senofilcon C compared to senofilcon A.

**Figure 2:**
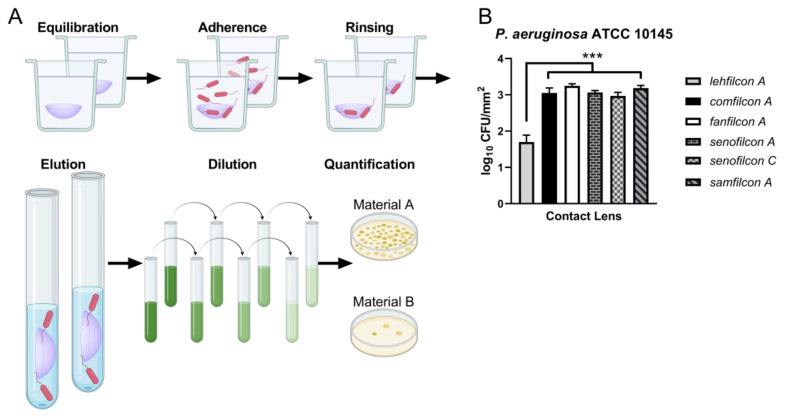
*P. aeruginosa* ATCC 10145 adheres poorly to lehfilcon A. (**A**) A depiction of the critical steps of the quantitative adhesion assay procedure used to compare contact lens materials. (**B**) Results of a quantitative adhesion assay of *P. aeruginosa* strain ATCC 10145 incubated with soft contact lenses (n = 6 individual contact lenses tested per material). The data are depicted as the average log density of colony forming units recovered from adhesion reactions (log10 CFU/mm^2^) ± S.D. *** represents *p* < 0.0005 for comparison to lehfilcon A.

To quantify the extent of the adhesion advantage of lehfilcon A seen by microscopy, we performed adhesion assays with unstained ATCC 10145, using live bacteria recovery from the contact lens surfaces as the readout (Figure 2). Lehfilcon A bound less (1.7 ± 0.2 log CFU/mm^2^) 10145 than comfilcon A (3.1 ± 0.1 log CFU/mm^2^), fanfilcon A (3.3 ± 0.1 log CFU/mm^2^), senofilcon A (3.1 ± 0.0 log CFU/mm^2^), senofilcon C (3.0 ± 0.1 log CFU/mm^2^), or samfilcon A (3.2 ± 0.1 log CFU/mm^2^). These results are statistically significant at α = 0.01 with *p* < 0.0005 for all pairwise comparisons of lehfilcon A and show that *P. aeruginosa* adheres to lehfilcon A at comparatively low densities.

**Figure 3:**
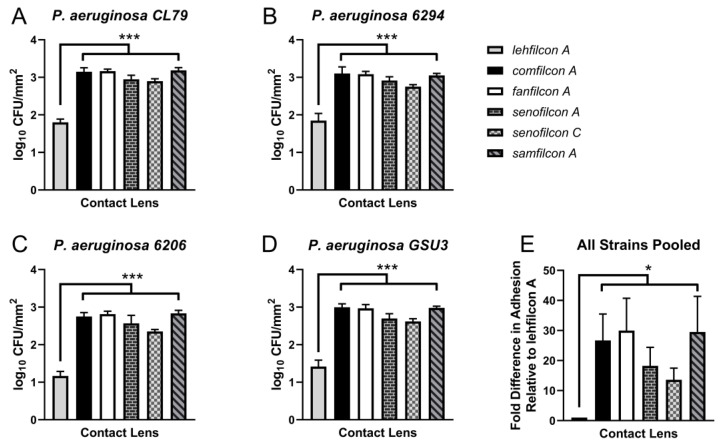
Lehfilcon A’s performance was consistent for various strains of *P. aeruginosa*. (**A**–**D**) Quantitative adhesion assays of *P. aeruginosa* strains GSU3 (**A**), CL79 (B), 6294 (**C**), and 6206 (**D**) exposed to soft contact lenses (n = 6 per lens type). The data are depicted as the average log density of CFUs recovered from adhesion reactions (log_10_ CFU/mm^2^) ± S.D. (**E**) Fold difference in *P. aeruginosa* adhesion for each lens material relative to lehfilcon A. The data depicts the pooled mean fold difference ± S.D. for all five quantified *P. aeruginosa* strains (10145, GSU3, CL79, 6294, and 6206). * represents *p* < 0.05, *** represents *p* < 0.0005 for comparison to lehfilcon A.

To determine if the trends in adhesion density seen for ATCC 10145 generalize to other strains, we performed quantitative adhesion assays for four additional clinically relevant *P. aeruginosa* strains (Figure 3). For *P. aeruginosa* strain CL79 (Figure 3A), lehfilcon A bound less (1.8 ± 0.1 log CFU/mm^2^; *p* < 0.0005 for all comparisons) than did comfilcon A (3.1 ± 0.1 log CFU/mm^2^), fanfilcon A (3.2 ± 0.0 log CFU/mm^2^), senofilcon A (2.9 ± 0.1 log CFU/mm^2^), senofilcon C (2.9 ± 0.0 log CFU/mm^2^), or samfilcon A (3.2 ± 0.1 log CFU/mm^2^).

Similarly, for strain 6294 (Figure 3B), lehfilcon A bound less (1.9 ± 0.2 log CFU/mm^2^; *p* < 0.0005 for all comparisons) than did comfilcon A (3.1 ± 0.2 log CFU/mm^2^), fanfilcon A (3.1 ± 0.1 log CFU/mm^2^), senofilcon A (2.9 ± 0.1 log CFU/mm^2^), senofilcon C (2.8 ± 0.0 log CFU/mm^2^), or samfilcon A (3.1 ± 0.0 log CFU/mm^2^). For strain 6206 (Figure 3C), lehfilcon A bound less (1.2 ± 0.1 log CFU/mm^2^; *p* < 0.0005 for all comparisons) than did comfilcon A (2.7 ± 0.1 log CFU/mm^2^), fanfilcon A (2.8 ± 0.1 log CFU/mm^2^), senofilcon A (2.6 ± 0.2 log CFU/mm^2^), senofilcon C (2.4 ± 0.0 log CFU/mm^2^), or samfilcon A (2.8 ± 0.1 log CFU/mm^2^). Finally, for strain GSU3 (Figure 3D), lehfilcon A permitted significantly less binding (1.4 ± 0.2 log CFU/mm^2^; *p* < 0.0005 for all comparisons) than did comfilcon A (3.0 ± 0.1 log CFU/mm^2^), fanfilcon A (3.0 ± 0.1 log CFU/mm^2^), senofilcon A (2.7 ± 0.1 log CFU/mm^2^), senofilcon C (2.6 ± 0.1 log CFU/mm^2^), or samfilcon A (3.0 ± 0.1 log CFU/mm^2^).

The average bacterial density among all five tested *P. aeruginosa* strains was calculated, and the fold difference in adhesion relative to the baseline set by lehfilcon A was calculated for each material (Figure 3E). The next best performing material after lehfilcon A, senofilcon C, bound 13.6 ± 3.9 times (*p* = 0.0019) more *P. aeruginosa* than did lehfilcon A. Of the other materials, comfilcon A bound 26.7 ± 8.8 times (*p* = 0.0028) more, fanfilcon A bound 30.0 ± 10.8 times (*p* = 0.0038) more, senofilcon A bound 18.2 ± 6.2 times (*p* = 0.0034) more, and samfilcon A bound 29.5 ± 11.8 times (*p* = 0.0057) more *P. aeruginosa* than lehfilcon A. These results demonstrate that lehfilcon A allowed less *P. aeruginosa* adherence than other contact lens materials, regardless of the strain used for testing. Interestingly, the trend of relatively good performance of senofilcon C was maintained among strains. This is in contrast to a study of lipid binding that observed relatively high total lipid binding of senofilcon C treated with an artificial-tears solution [58].

## 4. Discussion

The use of daily disposable contact lenses rose from 28% of contact lens users in 1998 to 63% of users as of 2022 [59]. This change in use habits has come at the expense of monthly contact lenses, such as those studied here. Daily disposable contact lenses are attractive to patients for their convenience, though they are generally more costly. Eyecare professionals appreciate daily disposable contact lenses for their relatively lower risk of complications [60,61]. To improve the competitiveness of monthly contact lenses, manufacturers would need to improve the design of these contact lenses, perhaps by mitigating risks associated with their use. Historically, there have been many improvements to contact lenses, including the transitions from rigid gas-permeable contact lenses to soft contact lenses and then to silicone hydrogel contact lenses. A potential area of improvement for next-generation contact lenses is the diminishment of microbially driven corneal inflammatory/infiltrative events (CIEs) associated with contact lens usage. CIEs occur when inflammatory signals promote leukocyte recruitment to the cornea, which may then cause irritation or tissue damage [62,63,64]. This can occur in instances where a microbe begins an infectious process, such as microbial keratitis. In such cases, a clinician may be able to isolate the causal organism by corneal scraping. However, CIEs can occur without the presence of a clinically identifiable pathogen, such as in cases of contact lens-induced acute red eye (CLARE) or contact lens peripheral ulcers (CLPUs). CLARE is a painful inflammatory disease of the cornea and conjunctiva associated with contamination of contact lenses by-products derived from Gram-negative bacteria, including *P. aeruginosa*, *S. marcescens*, *H. influenza*, and other organisms [65,66,67]. CLARE is believed to be precipitated by the presence of endotoxins released by these organisms, which recruit inflammatory cells [68]. CLPU is another infiltrative complication resulting in the loss of corneal epithelium [69,70]. CLPUs are not necessarily infectious but are associated with Gram-positive bacteria and their toxic products [71,72].

A current hypothesis in the field is that contact lens materials that can reduce microbial contamination may reduce CIEs. As a proof of concept of this hypothesis, a series of publications by Wilcox et al. detailed the results of in vitro experiments, animal model testing, and clinical outcomes for an experimental antimicrobial contact lens [73,74,75]. In these studies, the synthetic antimicrobial cationic peptide melimine was coated onto a contact lens to prevent microbial contamination of the material [73]. The authors observed melimine-concentration-dependent reductions in contact lens colonization of up to 92% for *P. aeruginosa* and 76% for *S. aureus*. In a follow-on in vivo study, melimine coating of contact lenses reduced the incidence of infiltrates from 50% to 13% in a guinea pig model of *P. aeruginosa*-dependent CLARE [74]. Similarly, the melimine lenses reduced epithelial defects from 27% to 9% in a *S. aureus* CLPU rabbit model [74]. A contact lens coated in the melimine-derived peptide, Mel-4, was assessed for CIE outcomes in human patients in a contralateral comparison study [75]. The study observed a 50–69% reduction in CIEs in patients using Mel-4 coated lenses for three months, relative to patients wearing the control contact lens. Combined, these studies offer strong evidence that advanced contact lens materials that prohibit contamination can reduce microbially driven contact lens complications.

Reduced microbial contamination of synthetic substrates has long been an infection prevention goal of the medical-device community. Two broad categories of polymer technologies have been employed to reduce the microbial burden on medical devices: antimicrobial surfaces that actively kill the microbes that encounter the device and anti-biofouling surfaces that reduce microbial binding to the device. These are still emerging strategies, and not all are likely to be compatible with consumer products. Important examples of antimicrobial surfaces include metal-impregnated polymers [76,77,78], drug-eluting composites [79,80], and coatings with antimicrobial peptides such as Mel-4 [73,81,82] and other cationic polymers [83]. Examples of anti-biofouling surfaces include zwitterionic polymers such as PMPC found on lehfilcon A, nanoscale topography [84,85], superhydrophobic coatings [86,87], and PEG-based coatings [88]. PMPC is a hydrophilic, biocompatible polymer displaying a zwitterionic phosphorylcholine group [89]. The PMPC on lehfilcon A is formed into a layer on top of a silicon hydrogel base material, yielding a hydrophilic and lubricious surface [46,47]. MPC has been previously demonstrated to confer resistance to protein adsorption to a variety of surfaces [90,91,92,93,94,95]. Similarly, the PMPC coating of lehfilcon A reduces protein adsorption relative to its base material [46,47]. PMPC is hypothesized to not interact strongly with proteins due to the formation of a stable water clathrate structure surrounding the PMPC chain [89,95].

Previously, we developed a fluorescent, live-cell, cell-surface, covalent labeling technique for studying a genetically intractable strain of *S. marcescens* by confocal microscopy [55]. We demonstrate the use of this technique for covalent labeling of *P. aeruginosa* with TAMRA-SE. Qualitatively, we observed that lehfilcon A bound relatively low levels of labeled *P. aeruginosa* strain ATCC 10145 (Figure 1). This successful implementation of TAMRA-SE staining for *P. aeruginosa* suggests the potential for broad use of this staining method for imaging live microorganisms in experimental settings that do not allow for genetic labeling with fluorescent proteins or transient labeling with non-covalent dyes. To quantitatively evaluate *P. aeruginosa* biofouling, we recovered and enumerated adhered organisms from each contact lens material. We observed low adhesion to lehfilcon A by unlabeled *P. aeruginosa* ATCC 10145 (Figure 2B). Our observations are in accordance with prior studies showing reduced *P. aeruginosa* binding to MPC coated steel plates [49] and our observations of *S. marcescens* interactions with lehfilcon A [55]. We observe that these results are independent of the strain used for testing (Figure 3), suggesting that the differences in adhesion between contact lenses are primarily due to differences in the contact lens materials. Most studies have used only a single microbial strain to evaluate adhesion to contact lenses, leaving considerable ambiguity as to whether their conclusions are dependent upon the material tested or the strain used for evaluation. We have performed a parallel analysis using five strains, including clinical isolates. Therefore, we can without ambiguity distinguish the role of the contact lens material in governing adhesion from variations caused by individual phenotypic behavior of a given strain. Interestingly, the general pattern of adhesion was remarkably consistent. Even minor trends in material performance were maintained among *P. aeruginosa* strains.

We observe that senofilcon C consistently trends towards lower levels of adhesion compared to senofilcon A, comfilcon A, fanfilcon A, and samfilcon A (Figure 3E). Senofilcon A, senofilcon C, and samfilcon A contain polyvinylpyrroliperformed (PVP) as a wetting agent that might be expected to perform an analogous function to PMPC on lehfilcon A, arguing that microbial adhesion to contact lens materials cannot necessarily be readily predicted and must be experimentally determined. Interestingly, a series of explorative studies pairing machine learning based prediction of microbial adhesion with high throughput synthesis and testing of polymer formulations found that material properties previously believed relevant had no value in predicting adhesion [96,97,98,99]. Further development of anti-biofouling medical devices would likely benefit from incorporating the kinds of high-throughput synthesis, testing, and computational tools now becoming available [100].

Our findings are relevant because CIEs, including contact lens-related keratitis and CLARE, are associated with Gram-negative pathogens, especially *P. aeruginosa* [2,7,9,10,65]. While keratitis can be caused by the microbiota resident to the eye, contact lenses are likely to function as a vector enabling the transfer of pathogenic microbes into the eye [31,101]. Indeed, SiHy lenses have been shown to allow more microbial adhesion to the contact lens surface compared to conventional hydrogel lenses, perhaps due to the relative hydrophobicity of SiHy lenses [45,102,103,104].

Contact lenses that intrinsically limit microbial contamination may be an effective approach to reducing CIEs when coupled with effective contact lens care solutions. Currently, we do not yet know if lehfilcon A will reduce CIEs in the hands of patients. However, the in vitro anti-biofouling properties of lehfilcon A are promising, and thus, we believe that our results may justify performing an observational study of lehfilcon A users to assess CIE outcomes.

## Data Availability

Not applicable.
